# Targeted Intervention Strategies for Seasonal Fluctuations in Daqu Microorganisms in Brewing

**DOI:** 10.3390/foods15091474

**Published:** 2026-04-23

**Authors:** Yanyan Tang, Yanbo Liu, Boya Shi, Nazir Ahmad Khan, Jian Xu, Chunmei Pan

**Affiliations:** 1 School of Life and Health Sciences, Hubei University of Technology, Wuhan 430068, China; 2 College of Life Sciences, Henan University of Animal Husbandry and Economy, Zhengzhou 450046, China; 3 Department of Animal Nutrition, The University of Agriculture, Peshawar 25130, Pakistan

**Keywords:** Baijiu Daqu, seasonal changes, microbial community, enzyme activity, regulation

## Abstract

Due to an insufficient understanding of the dynamic mechanisms underlying seasonal variations in Daqu fermentation quality, such as microbial community succession, enzyme activity, and metabolic regulation, precise control of Daqu quality and consistency across different seasons has not yet been achieved. Environmental factors, especially temperature and humidity, exert a significant influence on the microbial community structure and enzyme activity during the production process of Daqu. To this end, the recent research progress on changes in microbial community structure and succession, regulation of enzyme activity and metabolic pathways, and biosynthesis of flavor compounds in Daqu across different seasons was reviewed. Furthermore, we proposed strategies to leverage seasonal microbial patterns for precise control over Daqu production throughout varying seasons. This work aims to enhance the quality and consistency of Daqu across seasons and effectively improve the quality of Baijiu products.

## 1. Introduction

Baijiu is a kind of traditional alcoholic beverage produced in China mainly using grains for solid state fermentation with wine yeast, and then it undergoes distillation, aging, and blending. Brandy, whiskey, vodka, gin and rum are also considered as one of the 6 major distilled spirits around the world [1]. Because it has obvious regional features, the differences between baijiu are large; it is generally categorized according to different aromas: rice-aroma type, light-aroma type, strong-aroma type, and soy-sauce-aroma type [2].

**Figure 1:**
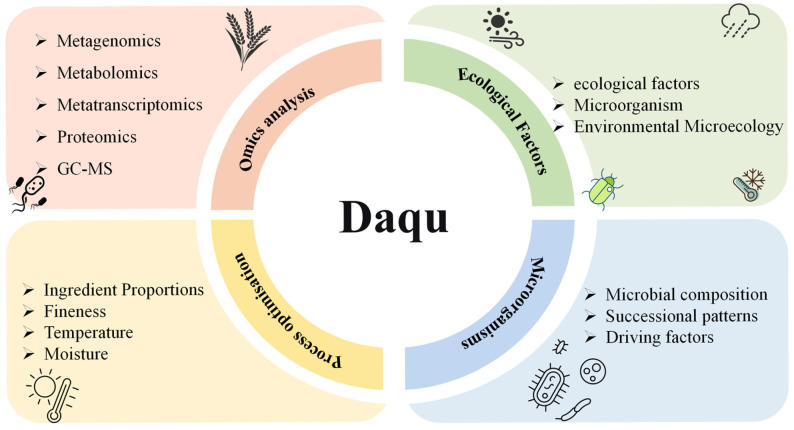
Multidimensional analysis of Daqu quality and factors affecting Daqu quality.

The market value of Chinese Baijiu is closely related to its overall quality, which is strongly influenced by the quality of Daqu, a key fermentation starter that directly determines both the yield and quality of the Baijiu produced. Daqu production involves traditional, open-air, solid-state fermentation processes, where process parameters, environmental conditions, and microbial composition directly impact Daqu quality [3]. The microbial community of Daqu determines the content and composition of aroma compounds, acids, and alcohols in Baijiu [4]. Numerous studies have shown that the fermentation of Baijiu is primarily achieved through the metabolic activities of microorganisms in the Daqu [5,6]. The fermentation system of Baijiu can be regarded as a self-regulating complex microbial ecosystem, where diverse microorganisms form a unique microbial community shaped by intricate interactions during fermentation. Microbial composition undergoes systematic succession throughout the fermentation process, influenced by environmental factors such as temperature and humidity. This highly complex, self-regulated succession of microbial communities drives the metabolic pathways that ultimately shape the flavor profile of Baijiu (Figure 1) [7,8,9]. This background provides strong impetus to conduct multidimensional analysis of the dynamic regulation of the Daqu microbial community, and devise strategies for enhancing Daqu quality to optimize the production of premium quality traditional Baijiu.

Environmental conditions are one of the primary exogenous drivers that shape microbial community structure under the open-air solid-state fermentation, and consequently determine the overall Daqu quality [10,11]. Next to changes in microbial communities, seasonal fluctuations also affect the physicochemical properties of the Daqu. To mitigate seasonal fluctuations in Daqu quality, traditional practices adjust several key parameters such as Daqu pile height and turning frequency to minimize seasonal impacts on the fermentation microenvironment [12,13]. Although this practice partially mitigates seasonal fluctuations in Daqu quality and their effects on Baijiu production, it often proves ineffective. Controlling microbial communities during Daqu production represent the fundamental solution to address seasonal variations in Daqu quality [10,14].

Microbial communities serve as the core functional carriers of Daqu, with their composition and metabolic activities determining the fermentation performance of Daqu. Enzyme activity, a key indicator of biological catalysis, not only regulates the fermentation process and the formation of flavor compounds, but also serve as a primary index for the underlying dynamic changes in microbial structure and function during the fermentation process [15]. Microorganisms serve as the primary source of enzymes, as such the types and quantities of enzymes directly reflect the composition of microbial community. Environmental factors indirectly influence enzyme synthesis and expression by regulating microbial growth and metabolism [16,17].

Different functional microorganisms exert synergistic or competitive effects during fermentation, forming complex metabolic networks. For instance, fungi secrete amylases to saccharify starch, while yeast relies on its alcohol-producing enzyme system to convert sugars into ethanol. This process is highly dependent on the orderly collaboration among microorganisms and the temporal regulation of enzyme production [18]. Therefore, developing precision control strategies for regulation of microbial community composition, especially the dynamics of their fluctuation to seasonal variations, is crucial for overcoming the limitations of traditional empirical methods and ensuring consistent Daqu quality. This approach represents a key pathway for enhancing fermentation performance and holds significant theoretical value in advancing Daqu production and Baijiu brewing techniques from traditional empirical practices towards precision-control systems.

## 2. Overview of Baijiu Daqu in Different Seasons

### 2.1. Definition, Classification, and Seasonal Characteristics of Daqu

**Figure 2:**
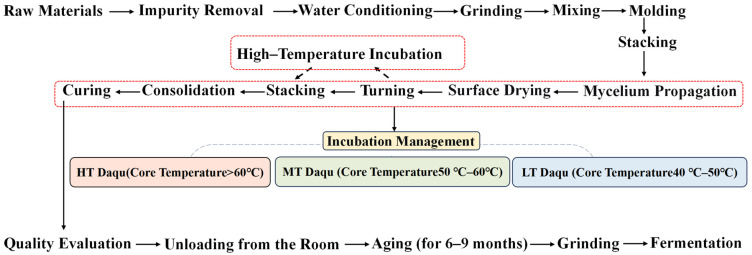
Simplified diagram of Baijiu Daqu production process.

Daqu, a unique saccharification and fermentation agent for Baijiu production, primarily uses raw grains such as wheat and barley as raw materials. Its manufacturing process involves grinding the raw grains, mixing them with water, pressing into shape, managing microbial cultivation, and storing them in the qu room. Daqu harness microorganisms from its natural inoculation environment to form complex microbial communities that sustain continuous fermentation. Enzymes produced through microbial metabolism, break-down macromolecules like starch and protein in the raw materials into diverse flavors producing end products. The resulting finished product, characterized by its block-like form, is termed “Daqu” (Figure 2) [8,19,20].

The microbial composition of Daqu varies with fermentation temperatures [21]. Based on temperature, Daqu can be classified into three types: high-temperature, medium-temperature, and low-temperature Daqu. High temperature Daqu, such as sauce-aroma Daqu, is produced under fermentation temperature of over 60 °C. Its characteristic microorganisms primarily consist of *Kroppenstedtia*, *Thermoascus*, *Thermomyces*, *Aspergillus*, and *Bacillus*. The high-temperature environment promotes the proliferation of thermophilic microorganisms like *Bacillus*. These microorganisms produce characteristic substances, such as melanin and melanoidins, through the Maillard reaction [6,22,23,24]. Medium-temperature Daqu (e.g., strong-aroma type), is produced at temperatures of 50–60 °C. Its characteristic microbial community includes *Weissella* and *Lactobacillus* [25,26,27,28]. Low-temperature Daqu (e.g., clear-aroma type) is produced at fermentation temperatures ranging from 40 to 50 °C. Its signature microbial community is primarily comprised of *Saccharomycopsis*, *Lactobacillus*, *Aspergillus*, *Bacillus*, and *Pantoea* [11,19,29]. Variation in microbial composition leads to distinct enzyme systems among Daqu types. For instance, thermophilic bacteria such as *Virgibacillus* and *Saccharopolyspora* exhibit higher relative abundance in high-temperature Daqu; however, their saccharification and liquefaction capacities are generally lower than those of medium-temperature Daqu. This disparity further affects the subsequent temperature curve of fermenting mash. For example the “Four High-temperature Characteristics” of jiangxiang-style mash, namely, high-temperature Daqu production, high-temperature stacking, high-temperature fermentation and high-temperature distillation, are shaped by the heat-tolerant enzyme systems of high-temperature Daqu [30,31].

The microecological functions of Daqu, including microbial community structure, enzyme activity, and flavor compound composition, exhibit distinct seasonal variation. The mild and humid climate in spring promotes the growth of *Lactobacillus* and *saccharomycetes*, while hot summer conditions accelerate the growth of thermophilic fungi such as *Thermoascus*, which enhances amylase activity and accelerates starch saccharification process. The stable temperature and humidity conditions in autumncreate favorable conditions for synergistic interaction between *Bacillus* and fungi of the genus *Aspergillus*, and their carbohydrate metabolic activity exhibits distinctive phased dynamics. The low temperatures in winter inhibit the overall microbial activity but promote the slow synthesis of aromatic substances such as vanillin. This unique metabolic mode sets the foundation for the clean and refreshing taste of Baijiu [10,32,33]. These season-induced variations in complex microbial communities further complicate the quality control across different seasons and increase the risk of batch-to-batch variations in Daqu.

### 2.2. Research Trends in Daqu Fermentation Across Different Seasons

In recent years, the rapid advancement of omics technologies has opened new avenues for studying Daqu across different seasons. The application of genomics, transcriptomics, proteomics, and metabolomics enables in-depth molecular analyses of microbial community composition, function, and interactions. These approaches can reveal the dynamic patterns of microbial communities, identify key functional components such as enzymes and flavor compounds, and elucidates their formation and regulatory mechanisms across different seasons and fermentation conditions [4,10,34]. Metagenomics is able to find out the function genes of the microorganisms of Daqu, like the ones about the starch breakdown and ester synthesis [35]. Metatranscriptomics could also show what genes are expressed at any time, which metabolism will be carried out when it is hot and humid [36]. Metabonomics can quantitatively analyze changes in flavor substances like alcohol, ester, acid and aldehyde etc., so it is possible to confirm the connection between geno and pheno types [36]. The introduction of omics technologies has expanded the depth and breadth of research into seasonal variation in Daqu, thereby offering robust scientific support for the modernization and improvement of traditional fermentation practices in Baijiu production.

Seasonal variations represent key external factors influencing the succession of microbial communities and flavor development during Daqu fermentation. To fully understand and mitigate seasonal variability in Daqu and Baijiu quality, research has evolved from traditional empirical descriptions toward an interdisciplinary approach integrating multi-omics, data science, and systems biology. This has led to an in-depth, mechanistic, and quantitative understanding of the Daqu fermentation process. Previous mechanistic research on Daqu fermentation primarily analyzed single environmental factors, focusing on the linear effects of independent variables such as temperature and humidity. This approach had limited capacity to elucidate complex phenomena like microbial community interactions and metabolic network coupling [11,16,37,38]. With the recent advancement in multi-omics technologies, it is possible to conduct multidimensional association analysis, integrating metagenomic, metabolomic, and other data and construct an “environment-microbiome-metabolite” association network [15]. These advancements have identified a critical window period during the early fermentation stage of Daqu. Environmental fluctuations during this phase can influence subsequent microbial succession and the accumulation of flavor compounds through temperature changes [16]. Under high-temperature conditions in summer, thermophilic bacterial communities significantly increase esterase gene expression, leading to a marked rise in production of flavor compounds such as ethyl acetate [39]. Although omics technology has become the main approach to revealing the seasonal rules of Daqu fermentation, current methods are still lacking and deserve a thorough examination and improvement [16]. Most studies are conducted at a single distillery and cannot be applied to multi-plant, open-mash, solid-state, large-scale malting environments; therefore, their findings cannot be directly applied to industrial production [40]. Secondly, most of the omics data are still correlational without causality and functionality validation and lack understanding about how microorganism, enzyme and flavor compound regulate [29]. Third, Different studies use different sequencing platform, analysis workflow, sample preparation method, so the data cannot be compared and also it is not possible to merge them [38,41]. With the advancement of omics technologies, research has undergone a fundamental shift in both understanding and regulating of the Daqu fermentation process. For example, metagenomics has revealed the functional potential of season-specific microbial communities, metatranscriptomics has elucidated the regulatory mechanisms of environmental factors on gene expression, and multi-omics integration has established direct links from microbial genotypes to flavor phenotypes [15,42,43]. Machine learning has further advanced the regulatory strategy by quantifying the nonlinear relationships between microbial succession and environmental factors through algorithms such as random forests and LSTM. For instance, models can analyze temperature change rates and microbial turnover rates to accurately predict final ester content and recommend dynamic fermentation adjustments. This propels Daqu production from experience-based control toward a new era of data-driven precision regulation [44,45]. From an application perspective, Daqu research is transitioning beyond quality stabilization toward the integration of green production and high-value utilization strategies. Recently progress has been made in green manufacturing technologies. For example, employing multi-strain combinations instead of single strains effectively achieves efficient degradation of lignocellulose in bran. This provides a new green circular pathway for developing high-quality second generation fermented products [46,47]. In a high-value development, based on the spatio-temporal correlation patterns between microbial communities and flavor profiles, targeted inoculation of functional bacterial strains were able to increase ethyl hexanoate content, allowing precise design of distinctive flavors [15]. With the successful application of machine learning models in flavor prediction, the Daqu industry is accelerating its transformation from traditional experience-based production to digital data-driven precision design, providing an innovative paradigm for the sustainable development of the entire fermented food sector [48].

In summary, Daqu research has established a clear development trajectory: in mechanistic studies, achieving a leap from observation of superficial phenomena to the elucidation of fundamental principles; in control technologies, transitioning from experience-dependent parameter optimization to data-driven precision regulation; and in applied research, extending beyond quality stabilization toward integrated innovation in green, low-carbon, and high-value products. Looking ahead, the deep integration of multimodal data with mechanistic models will enable cross-seasonal, predictable precision regulation, the core direction for advancing modern brewing processes.

## 3. Functional Microorganisms in Baijiu Daqu Across Different Seasons

### 3.1. Definition and Research Methods of Functional Microorganisms

Daqu harbors a very diverse microbial community, comprising of Bacteria, Yeast, Fungi. The microbial structure and function exhibit both spatial heterogeneity and dynamic temporal evolution. Through complex metabolic interactions, these microorganisms form intricate networks that drive the core ecological processes such as carbon and nitrogen cycling, organic matter degradation, and energy transfer [49,50]. Functional microorganisms in Daqu carries out specific functions in the fermentation process, such as enzymes, ester and acid production. By secreting enzymes or metabolic products, these functional microorganisms directly contribute to raw material degradation and flavor compound synthesis, serving as the core biological factors determining Baijiu quality [51,52]. As *Saccharomyces* and *Lactobacillus* respectively dominate the alcoholic fermentation and acidification stages, their functional division reflects the temporal collaboration within microbial communities [53].

The characterization of functional microorganisms requires integration of genetic lineage and metabolic characteristics. Currently, screening and identification techniques for functional microorganisms have evolved from traditional culture-based methods to multi-omics integrated strategies. Next to that, metabolomics can be employed to track ester synthesis pathways, such as fatty acid β-oxidation [54,55]. Metagenomics can reveal associations between functional genes and microorganisms. For example, by designing degenerate primers targeting zinc-dependent long-chain alcohol dehydrogenase genes, we can specifically track the distribution patterns of higher alcohol-producing microorganisms such as *Bacillus* and *Aspergillus* [56,57]. Macrotranscriptomics can be utilized to reveal the gene expression regulation mechanisms and metabolic functions of functional microorganisms in Daqu, such as *Bacillus velezensis*. Biological disturbance significantly alters the core microbial community of Daqu from *Aspergillus chevalieri* to *Paecilomyces varioti*, while concurrently activating starch metabolism and pyrazine biosynthesis pathways, resulting in a 24.5% increase in pyrazine content [43]. By quantifying the contribution of environmental factors and raw materials to microbial community composition using SourceTracker, the theoretical basis for targeted regulation can be established [58]. The application of these methods has enabled the analytical correlation between functional microorganisms and their metabolites. For example, *Klebsiella* and *Saccharopolyspora* have been confirmed to exhibit a positive correlation with vanillin biosynthesis [59]. Future research may integrate multi-omics technologies, such as metagenomics and metabolomics, to deeply analyze microbial functional networks. By combining traditional processes with modern techniques, targeted microbial communities can be constructed to achieve controllable flavor profiles and production stability in Daqu fermentation.

### 3.2. Seasonal Variations in Functional Microorganisms of Baijiu Daqu

The core functional microorganisms in Daqu primarily consist of bacterial species such as *Bacillus* and *Kroppenstedtia*, while fungi are represented by *Thermoascus* and *Saccharomycopsis*. Daqu has different microbes doing different things. *Aspergillus* is the main mold which can secrete a lot of hydrolytic enzymes like amylase and saccharifying enzymes. And it degrades starch into fermentable sugars which are the substrate for fermentation as well it is involved with creating aromatic precursors [4,60]. *Bacillus* can produce proteases, esterases, and it is also an important functional bacterium. By means of metabolism, it synthesizes typical aromatic flavor compounds like tetramethylpyrazine, ethyl acetoxy compound, ethyl hexanoate etc., bestowing the unique roasted and soy sauce scents upon Daqu [61,62]. Yeast mainly takes part in the formation of the higher alcohols and esters which add more layers of alcohol and fruit to the spirit. It can’t control the process of alcoholic fermentation but it has some influence on how complex your flavor profile is [63]. Through synergistic interactions, these functional microorganisms form Daqu’s unique enzyme system and flavor precursor compounds [24]. Additionally, the community structure and metabolic functions of functional microorganisms exhibit significant seasonal variations (Table 1). During summer, the thermophilic genus *Bacillus* dominates, with its relative abundance reaching 2 to 3 times higher than that observed in winter. These thermophilic microorganisms effectively maintain cellular homeostasis in high-temperature environments through ecologically developed sophisticated molecular mechanisms, such as by upregulating heat shock protein expression and adjusting cell membrane fatty acid composition. In winter, the genus *Lentibacillus* becomes predominant microbial group. These cold-loving bacteria maintain basal metabolic activity in low-temperature environments through strategies such as synthesizing cryoprotectants and adjusting the thermal properties of enzyme systems. However, overall microbial diversity decreases during winter. Spring and autumn serve as transitional periods, characterized by the coexistence and functional equilibrium of yeasts and *Aspergillus* [3,10,11,64].

Next to community structure, seasonal variations in temperature, humidity and light exposure can directly influence the enzymatic activity of functional microbial communities in Daqu, thereby determining Baijiu brewing efficiency and flavor compounds. During the saccharification and piling process of summer Daqu, temperature and acidity often exhibit significant fluctuations, causing α-amylase and saccharifying enzyme activities to exhibit an “early peak followed by rapid decline.” This phenomenon is closely related to the microbial community structure of summer Daqu, where *Bacillus* species with heat-tolerant characteristics dominate. *Bacillus* not only secretes amylase and protease to facilitate raw material degradation but also synthesizes pyrazine compounds like tetramethylpyrazine through non-enzymatic pathways, enriching the flavor complexity of Baijiu [65]. The higher mold content in summer Daqu is typically associated with increased amylase activity, thereby enhancing saccharification efficiency and reducing esterase yield [57]. On the other hand, during summer, ester compounds such as ethyl acetate and ethyl caproate reach peak levels in Daqu metabolites. In southern production areas, the synthesis rate of ethyl acetate in summer can be up to 1.8 times higher than that of northern areas during the same period. However, this metabolic advantage is often accompanied by a relative decrease in esterase production [21,66,67]. Cellulase and amyloglucanase are the key functional enzymes during spring, resulting in relatively higher levels of aldehydes and ketones among the Daqu metabolites. In contrast, autumn Daqu exhibits a more balanced composition of enzymes, resulting in an optimal equilibrium among protease, amylase, and esterase activities. This balance manifests in rich diversity of complex esters that give rise to its unique flavor profile [11,68]. Daqu fermentation during winter is predominantly carried out by *Saccharomycopsis*. Its metabolic characteristics favor the synthesis of ethanol and characteristic alcoholic flavor compounds such as n-propanol and amyl alcohol [10]. Specific microorganisms such as *Bacillus* exhibit a significant correlation with the production of pyrazine compounds, and the strength of this association shows marked seasonal variation [48,69]. This further confirms that seasonal changes in the content and composition of Daqu metabolites is predominantly regulated by the season-induced changes in functional microbial communities.

Microbial functional redundancy serves as a key mechanism for maintaining brewing stability. During the high-temperature phase, the synergistic interaction between *Bacillus* and thermophilic fungi compensates for the functional loss of mesophilic microbial communities. Whereas, the symbiotic network of *Lactobacillus* and *Saccharomyces* ensures the continuous functioning of basal metabolism during low-temperature phase [50]. Extreme weather events can disrupt this balance, and the recovery of microbial communities can be accelerated by inoculation of functional microbes or adjusting the fermentation process [70].

In summary, environmental conditions directly influence microbial cells through physical and chemical forces. The Daqu microbial communities are ultimately shaped via competition and functional redundancy in response to seasonal fluctuations. These community level adjustments in microbial composition and metabolic networks drive brewing functions and maintain product quality. Current research employs metagenomics to decipher microbial-metabolite association networks and identify targets for artificial regulation. For instance, adding functional microorganisms can significantly enhance the content of flavor compounds in Daqu, enabling targeted flavor enhancement [15,43,62,63]. These findings also revealed the intrinsic patterns of seasonal succession of functional microorganisms, providing a solid theoretical foundation for artificially regulating environmental parameters to optimize traditional processes, and consistently produce high quality Baijiu.

**Table 1:** The characteristic microbial groups of Daqu in different seasons and research methods.

Season	Research Methods	Characteristic Microbial Community Composition	References
Spring	Illumina HiSeq sequencing	*Bacillus*, *Oceanobacillus*, *Pantoea*, *Lentibacillus*, *Kroppenstedtia*, *Weissella*, *Enterobacter*, *Staphylococcus*, *Pseudogracilibacillus*, *Esherichia-Shiegella*, *Lactococcus*, *Thermoactinomyces*	[10]
	Illumina HiSeq sequencing	*Pichia*, *Bacillus*, *Thermoascus*, *Lactobacillus*, *Pantoea*, *Klebsiella*, *Rhizomucor*, *Weissella*, *Rhizopus*, *Leuconostoc*, *Pediococcus*	[71]
	high hroughput sequencing	*Bacillus*, *Weissella*, *Thermomyces*, *Apiotrichum*, *Acinetobacter*, *Clavispora*, *Geotrichum*, *Issatchenkia*	[14]
	Metagenome	*Bacillus*, *Aspergillus*, *Oceanobacillus*, *Geobacillus*, *Schizosaccharomyces*, *Neurospora*, *Listeria*, *Anoxybacillus*, *Saccharomyces*, *Alkalihalobacillus*, *Virgibacillus*	[11]
Summer	Illumina HiSeq sequencing	*Bacillus*, *Kroppenstedtia*, *Enterobacter*, *Pantoea*, *Oceanobacillus*, *Lentibacillus*, *Staphylococcus*, *Pseudogracilibacillus*,	[10]
	metagenomic and metaproteomic	*Lichtheimia*, *Rhizopus*, *Lactobacillus*, *Aspergillus*, *Streptomyces*, *Saccharopolyspora*, *Leuconostoc*, *Pichia*, *Weissella*, *Staphylococcus*, *Lactobacillus*, *Saccharomycopsis*, *Saccharopolyspora*, *Leuconostoc*, *Weissella*, *Staphylococcus*, *Pantoea* *Pediococcus*, *Bacillus*, *Acetobacter*	[33]
	High throughput sequencing	*Clavispora*, *Fusarium*, *Geotrichum*, *Bacillus*, *Saccharopolyspora*, *Thermoascus*, *Thermomyces*, *Aspergillus*	[14]
	Illumina HiSeq sequencing	*Thermoascus*, *Weissella*, *Sliene*, *Weissella*, *Lactobacillus*, *Alternaria*, *Rhizopus*, *Aspergillus*, *Bacillus*, *Saccharopolyspora*, *Pediococcus*, *Pichia*	[71]
Summer	Metagenome	*Anoxybacillus*, *Alkalihalobacillus*, *Thermomyces*, *Thermoascus*, *Rhizopus*	[11]
Autumn	Illumina HiSeq sequencing	*Bacillus*, *Oceanobacillus*, *Pantoea*, *Lentibacillus*, *Kroppenstedtia*, *Weissella*, *Enterobacter*, *Staphylococcus*, *Pseudogracilibacillus*, *Esherichia-Shiegella*, *Lactococcus*, *Thermoactinomyces*	[10]
	metagenomic and metaproteomic	*Lichtheimia*, *Rhizopus*, *Lactobacillus*, *Aspergillus*, *Streptomyces*, *Saccharopolyspora*, *Leuconostoc*, *Pichia*, *Weissella*, *Staphylococcus*, *Lactobacillus*, *Saccharomycopsis*, *Saccharopolyspora*, *Leuconostoc*, *Weissella*, *Staphylococcus*, *Pantoea* *Pediococcus*, *Bacillus*, *Acetobacter*	[33]
	High throughput sequencing	*Pseudomonas*, *Clavispora*, *Geotrichum*, *Rhodococcus*, *Bacillus*, *Weissella*, *Thermoascus*, *Thermomyces*, *Aspergillus*	[14]
	Metagenome	*Bacillus*, *Aspergillus*, *Oceanobacillus*, *Saccharomyces*, *Schizosaccharomyces*, *Neurospora*, *Thermotoge*, *Geobacillus*, *Virgibacillus*, *Listeria*	[11]
Winter	Illumina HiSeq sequencing	*Bacillus*, *Oceanobacillus*, *Lentibacillus*, *Pantoed*, *Kroppenstedtia*, *Enterobacter*, *Staphylococcus*, *Pseudogracilibacillus*, *Pseudomonas*	[10]
	metagenomic and metaproteomic	*Lichtheimia*, *Rhizopus*, *Lactobacillus*, *Aspergillus*, *Streptomyces*, *Saccharopolyspora*, *Leuconostoc*, *Pichia*, *Weissella*, *Staphylococcus*	[33]
	High throughput sequencing	*Geotrichum*, *Cyberlindnera*, *Bacillus*, *Saccharopolyspora*, *Thermoascus*, *Thermomyces*, *Aspergillus*, *Lactobacillus*	[14]
	Metagenome	*Bacillus*, *Aspergillus*, *Oceanobacillus*, *Saccharomyces*, *Schizosaccharomyces*, *Neurospora*, *Geobacillus*, *Virgibacillus*	[11]

## 4. Seasonal Characteristics and Applications of Baijiu Daqu Styles

### 4.1. Physicochemical Properties and Microbial Communities of Baijiu Daqu in Different Seasons

Daqu, serving as the core fermentation agent, functions both as a microbial carrier and an enzyme reservoir. Seasonal fluctuations in its quality affect the microbial composition spectrum and the content and composition of metabolic products, thereby altering the formation of Baijiu’s flavor profile [25,29,64]. The growth, metabolism and community of microorganism is influenced by many factors such as physical and chemical. As for physical factors, they contain the ambient temperature, relative humidity, content of oxygen, increase in water activity, and mechanical force etc. [72,73]. Chemical factor contains pH value, acid, the composition of carbon source and nitrogen source, and concentration of osmotic pressure, accumulated metabolic product [74]. Physicochemical factors can influence enzyme activities inside microbial cells, the firmness of cell membrane, as well as the extent of material transportation; thus, it results in different kinds and amounts of microbes in the large grain malt [75]. Seasonal variations cause distinct differences in the physicochemical properties of Daqu. High temperatures in summer accelerate water evaporation from Daqu, generally resulting in greater acidity levels [76]. During winter, low temperatures slow down the fermentation rate of Daqu, reducing the activity of amylase and protease [77]. These seasonal variations further drive the ecological succession of microbial communities. Thermophilic bacteria such as *Bacillus* and *Thermomyces* significantly dominate the microbial community during summer, whereas *Saccharomyces* and *Lactobacillus* are predominant during winter. Through their metabolic activities, these microorganisms produce distinct flavor precursors, shaping the unique flavor characteristics of Daqu across different seasons [78,79]. More importantly, the seasonal succession of microbial communities regulates the direction of functional metabolic pathways. Under high summer temperatures, microbial diversity decreases, but thermotolerant strains enhance saccharification and protein degradation pathways, promoting ester synthesis. Spring and autumn’s milder climates better sustain microbial diversity. The symbiotic relationship between *Saccharomyces* and molds synergistically produces complex alcohols and ketones. In spring-brewed Daqu, *Candida* enrichment correlates positively with polyphenolic accumulation-compounds that confer Baijiu’s antioxidant properties while generating toasty aromas through Maillard reaction derivatives [10,11,80].

The interaction between environmental factors and microbial communities is the core driving force behind Daqu quality. Variations in temperature and humidity, on one hand, regulate microbial growth rates, and on the other hand, indirectly influence the spatial distribution of aerobic and anaerobic bacteria by altering the core structure of the koji mold substrate. For instance, high humidity during the rainy season inhibits the growth of *Aspergillus*, and promotes the proliferation of facultative anaerobic bacteria such as *Pediococcus*, leading to organic acid accumulation and a decrease in pH. Seasonal differences in fermentation cycles also result in gradient changes in microbial metabolites, long-term fermentation in winter is more conducive to the production of flavor compounds with slow release characteristics, such as higher alcohols [9,81,82]. This spatiotemporal heterogeneity poses challenges for standardized Daqu production, while also providing scientific rationale for achieving targeted regulation of microbial communities.

### 4.2. Differences in Flavor Components and Their Formation Mechanisms

Daqu’s flavor is represented in the flavor by the ester, organic acid, alcohol, aldehyde, ketone, aromatic compound. The kinds as well as concentrations of those metabolic products will impact the aroma of the liquor. It has a relationship with the structure of the microbial group and enzyme activity, it is also greatly influenced by season and environment [20,26,83,84]. The seasonal variations in flavor primarily originates from the differences in the content and composition of volatile compounds such as esters, alcohols, and ketones. Environmental temperature and humidity drive the succession of microbial communities, and the differentiation of metabolic pathways and fermentation end products [47,85]. Research indicates that high-temperature Daqu contains higher levels of Maillard reaction products such as furans and pyrazine compounds compared to low-temperature Daqu. This phenomenon is closely associated with the enrichment of heat-tolerant microorganisms like *Kroppenstedtia* and *Monascus* [86,87]. The Maillard reaction becomes active under high-temperature conditions, generating volatile small molecules such as furfural and macromolecular products like melanoidins. These components collectively form the characteristic flavor profile of sauce-aroma Baijiu [53,88].

Seasonal changes in microbial metabolic pathways shape the final flavor profiles. During high-temperature phases, fungal communities such as *Aspergillus* and *Penicillium* relying on highly expressed carbohydrate-degrading enzymes to promote starch degradation, and drive the final conversion to aromatic compounds that form the final unique flavor precursors [39,89]. *Lactobacillus* and *Acetobacter* metabolism proceed predominantly through glycolysis pathway that generates organic acids and ester precursors, *Saccharomyces* is the main microorganism carrying out alcoholic fermentation, converting sugars that can be fermented to produce ethanol and involved in the production of some flavor compounds like higher alcohol and ester. It’s very sensitive to the seasonal temperature changes which makes it one of main reasons causing the seasonality difference in fermentation performance and flavor. Although *Pichia* doesn’t control alcoholic fermentation, it still helps make aromatic compounds like isopentanol and phenethyl alcohol; keeps community life going during times of strain on the environment; works together with other tiny creatures to influence how mixed up things are in making alcohol [90,91]. The proportions of these metabolites vary across different seasons in the Daqu, leading to seasonal flavor differences. The dynamic interaction between environmental factors and microbial activity amplifies the diversity in flavor composition of traditional Baijiu [92,93,94]. This multi-layered spatiotemporal heterogeneity results in distinct seasonal variation in the flavor compounds of Daqu.

### 4.3. The Correlation Between Seasonal Variation in the Stylistic Characteristics Baijiu Daqu and Baijiu

The dynamic evolution of Daqu quality across seasons forms foundation for a complex balance of enzymes system that drive the unique Baijiu flavor profile. Amylase converts grain starch into fermentable sugars, cellulase breaks down cellulose to release aromatic precursors, and esterase catalyzes ester synthesis. Daqu quality determines the rate and extent of these biochemical reactions, which collectively constitute the core pathway for Baijiu flavor formation [4,15]. Since the structure and activity of microbial communities vary significantly with the seasons, the formation and content of flavor metabolites such as esters, alcohols, and acids are also affected; therefore, when fermentation is conducted in different seasons, the flavor characteristics of baijiu do indeed differ [95,96]. Daqu produced in different seasons exhibits distinct enzymatic activity profiles due to variations in microbial communities. Summer Daqu features enhanced thermophilic-dominated high-temperature amylase activity, while winter Daqu is enriched with cold-adapted enzyme systems. These dynamic shifts result in seasonal variations in the final Baijiu’s flavor profile [25,33,97]. Seasonal changes regulate the composition of microbial communities through environmental parameters such as temperature and humidity, thereby influencing metabolic functions. This leads to the redistribution of key metabolic pathways, including starch hydrolysis and ester synthesis, ultimately determining the composition of flavor precursors in Baijiu [98,99]. The α diversity index of microorganisms in Daqu exhibits significant seasonal fluctuations, with the high humidity of the rainy season typically leading to a marked increase in species evenness [8,26]. Metagenomic analysis reveals that gene expression levels associated with the glycolytic pathway (EMP) and the tricarboxylic acid (TCA) cycle in summer Daqu reach their annual peak, effectively promoting the synthesis of core flavor compounds such as ethyl hexanoate and ethyl acetate [100,101]. In contrast, low winter temperatures inhibit the ethanol metabolic pathway of yeast, reducing overall fermentation efficiency by 15 to 20% and leading to the accumulation of undesirable flavor compounds such as phenethyl alcohol. These metabolic differences are particularly pronounced in sauce-aroma-type Daqu. For instance, Daqu produced in autumn typically exhibits higher esterification capacity, with ethyl acetate content reaching 2–3 times greater than that of summer production [102,103].

Recently, integrated analysis of multi-omics data further confirms a strong correlation between climate parameters and flavor compounds in baijiu. A significant, strong positive correlation between environmental temperature and esterase activity (R^2^ = 0.82) has been reported [38,104]. While high humidity conditions tend to trigger abnormal accumulation of aldehyde compounds. Within the strong-aroma Daqu system, the abundance of *Bacillus marinus* and other esters-synthesizing microbial communities reaches its annual peak during spring. This characteristic is clearly correlated with the enhanced cellar aroma intensity of the base liquor [105]. PICRUSt functional prediction analysis revealed that seasonal variations can affect more than 70% of microbial metabolic pathways. The metabolic activity of the acetyl-CoA synthesis pathway in summer Daqu was approximately 1.8 times greater than that of winter. From the perspective of metabolic flux, this difference explains the intrinsic mechanism underlying the seasonal fluctuations of typical caramel-like aroma compounds in Jiangxiang Baijiu [10].

Research on microbial functional validation remains significantly inadequate. Although preliminary models linking climatic factors, microbial communities, and metabolic end products have been established, the practical effectiveness of artificially regulating microbial communities to achieve flavor stability remains insufficient. Future research should integrate synthetic biology approaches to engineer seasonally dominant strains, e.g., thermophilic streptococci in summer or lactic acid bacteria in winter, for targeted modification to maintain Daqu quality during seasonal fluctuations. Furthermore, the mechanisms by which extreme climate events during winter and summer such as high-temperature droughts impact fermentation stability require deeper elucidation, necessitating molecular-level identification of specific disruption pathways affecting core microbial community interaction networks.

## 5. Multi-Faceted Regulation of Baijiu Daqu Application Strategies Across Different Seasons

**Figure 3:**
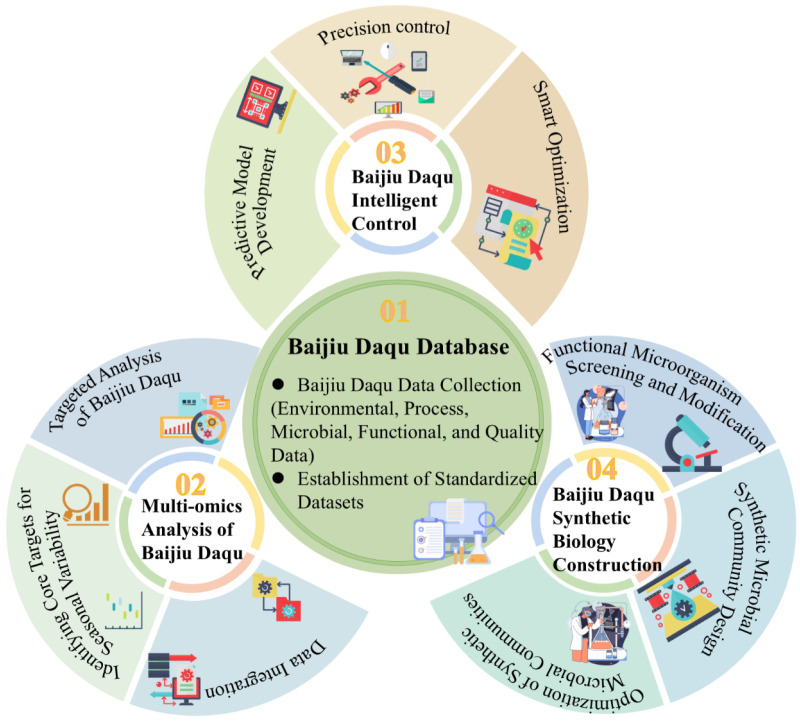
Baijiu Daqu Quality Adjustment Strategy.

Seasonal variability in Daqu quality has been widely reported, however, research on regulation of Daqu quality under different seasonal conditions remains fragmented and insufficient. First, the molecular mechanisms underlying the interaction between environmental parameters and microbial communities remain incompletely elucidated, particularly how temperature and humidity fluctuations influence functional gene expression through signaling pathways. Second, industrial production lacks standardized process parameters tailored to different seasons, resulting in inconsistent Daqu quality. Furthermore, traditional detection methods struggle to capture the synergistic changes in enzyme activity and metabolic end products during fermentation in real time, limiting the precision of dynamic regulation strategies. These bottlenecks urgently require breakthroughs through the establishment of a multi-level regulatory system. This system should encompass a comprehensive database covering data collection, mechanism analysis, intelligent prediction and precise intervention (Figure 3).

During the data collection phase, environmental data such as temperature, humidity, ventilation volume, and light exposure duration in the *Aspergillus oryzae* production workshop needs to be gathered. Process data including raw material ratios, grinding fineness, and water addition volume are collected. Microbial data encompassing the quantity of functional microorganisms at different fermentation stages of Daqu are recorded. Functional and quality data such as key enzyme activity, flavor compound content, and sensory evaluation of Daqu are also obtained to construct a comprehensive, multi-dimensional database covering the entire baijiu Daqu production chain. By defining abundance thresholds for core microbial communities (e.g., *Aspergillus*, *yeasts*) and functional gene expression levels, seasonal fluctuation, early warning indicators can be established. The integration of multi-omics technologies can provide novel insights into deciphering the relationship between Daqu microbial communities and functional mechanisms. Metabolomics and proteomics can be employed to analyze environmental effects on Daqu microorganisms, revealing microbial enrichment during environmental changes and how these microbes influence ethanol fermentation efficiency and flavor compound synthesis through enhanced enzyme activity and differential expression in flavor compound degradation pathways. Combined metabolomics and metagenomics analysis can reveal dynamic adjustments in microbial functional pathways under seasonal fluctuations. This can provide cross-system validation for metabolic interactions between alcohol-producing and aroma-producing bacteria in Daqu, indicating that the environment-microbe-function linkage mechanism possesses a universal ecological foundation. Consequently, constructing synthetic core microbial communities can provide experimental basis for multi-omics data-driven precision screening of functional microbial communities. During data integration, Python 3.12 can be employed to consolidate multi-source datasets, with correlation analysis preliminarily identifying key indicators affecting Daqu stability. Subsequently, functional redundancy can be leveraged to understand microbial community reconstruction patterns following environmental stress. Finally, integrating machine learning with ecological modeling, can be incorporate contextual variables such as geographical variations within the factory and raw material batches into the key factor screening process. This approach provides molecular-level screening criteria for regulating functional bacteria to adapt to seasonal changes within the environment-microbe interaction network.

After data collection, temperature-sensitive functional genes are identified first using metabolic pathway annotation. Next, research framework can be established for studying Daqu microbial communities across different seasons according to community hierarchy levels. Finally, based on the shaft system theory developed by previous researchers [106], predictive models can be constructed to link environmental parameters to core microbial communities and fermentation performance. For quality prediction, predictive frameworks can be developed for key Daqu quality indicators using various machine learning algorithms: Classic algorithms like XGBoost and Random Forest can be employed, incorporating seasonal variations in Daqu production, workshop environmental parameters, and process operation data as input variables. This can enable precise prediction of Daqu enzyme activity, flavor compound production, and microbial community similarity to optimal seasonal Daqu communities. Based on CNN-LSTM deep learning models, time-series monitoring data can be integrated throughout the entire fermentation cycle to perform predictive assessments of Daqu quality grades. Finally, model parameters from mature Daqu workshops in core production areas like Henan can be transferred to newly established workshops or cross-seasonal production facilities, reducing data collection costs and training cycles in new scenarios. In the dimension of precise process control, fine-tuning system parameters can enable dynamic adaptation and optimization of Daqu production parameters. For instance, during winter, workshop temperatures are regulated at 28–30 °C while raw material water addition increases by 5–8%; in summer, workshop humidity is reduced to 60–65% and fermentation cycles shortened by 3 to 5 days. The unsupervised learning model based on autoencoders enables intelligent early warning for abnormal fermentation states. Upon detecting anomalies like excessive mold proliferation in summer or sluggish enzyme activity growth in winter, it automatically triggers interventions such as adjusting koji-turning frequency or targeted supplementation of functional microorganisms. Simultaneously, by integrating real-time seasonal environmental data, the system can intelligently generate dynamic adjustment plans for raw material ratios and fermentation parameters, providing technical support for the precise and intelligent implementation of koji-making processes.

Closed-loop optimization of process parameters will require establishing quantitative mapping relationships between environment, microorganisms, and function. By integrating hyperspectral imaging with support vector regression, a non-destructive detection model for Daqu moisture content can be developed, enabling real-time feedback of key parameters during fermentation. This fusion of sensing technology and AI algorithms provides a data acquisition foundation for process control. Researchers inoculating Acinetobacter strains during cigar tobacco fermentation significantly increased flavor compounds like nicotinoids, demonstrating that targeted regulation of exogenous microbial communities can optimize metabolic networks [107]. This finding provides experimental evidence for constructing synthetic microbial communities resistant to seasonal fluctuations. DGGE analysis revealed comparable microbial Shannon indices between mechanically prepared and manually prepared Daqu [108], suggesting that standardized microbial inoculation may serve as an effective approach to overcome seasonal dependency. Current research must still address critical issues such as core microbial community selection, niche allocation, and functional maintenance under environmental stress.

Through integrated analysis of ecological models and multi-omics data, the regulatory weights of environmental factors on microbial communities and metabolic processes can be quantified in Daqu fermentation. First, drawing on machine learning modeling approaches, an associative model linking environmental factors, metabolites, and microbial distribution needs to be constructed. This model incorporates key parameters such as temperature and humidity from different seasonal fermentation environments, along with metabolite concentration data (e.g., lactic acid, ethanol) from corresponding systems. The model analyze how environmental parameters cause variations in synthetic microbial distribution and validated the model’s consistency with natural community heat stress response patterns. Second, a pressure gradient culture system simulating seasonal transitions can be established to track metabolic network module reorganization in microbial communities. The focus is on observing the activation status of extracellular enzyme secretion pathways in cold-tolerant bacteria under low-temperature conditions, elucidating the biological basis of microbial community functional plasticity. Concurrently, bioprocess intensification techniques can be introduced to evaluate the improvement effects on fermentation cycle and flavor consistency by directing metabolic flux allocation in core microbial communities. Finally, within synthetic microbial communities, we can concurrently conduct species composition profiling, co-occurrence network analysis, and gene functional annotation to overcome the limitations of single-species composition data in predicting functional outputs. Drawing on synthetic microbiome construction methods proposed in probiotic research [109], we can achieve stable regulation of the core microbial community in Daqu through precise screening of functional strains and community assembly principles. Key functional microbial communities are identified through multi-omics data analysis. Leveraging a comprehensive database spanning environmental parameters, microbiome, and metabolites, dynamic modeling can enable a breakthrough from correlation analysis to causal mechanism elucidation. This can ultimately provide methodological support and theoretical foundations for establishing an intelligent regulation system for Daqu production.

Future development should focus on technological integration and standardization. At the mechanistic level, combining multi-omics technologies with in situ detection methods can reveal the spatial distribution, metabolic networks, and functional expression patterns of microorganisms in solid-state fermentation. Technologically, promoting the integration of functional microbial screening, rapid detection, and intelligent modeling can achieve precise prediction and dynamic regulation of fermentation processes. Process-wise, standardized integrated system for parameter optimization, online monitoring, and risk control can be established. This will drive the transformation of Daqu production from experience-driven to data-driven approaches, enabling the precision, standardization, and intelligent evolution of traditional brewing techniques.

## 6. Conclusions and Outlook

Daqu’s production is a kind of open-fermentation production which is greatly influenced by the environment. Seasonal change has long changed the microbial population, enzyme function and compound flavor which had great influence on the quality stabilities of daqu. This paper gives an overview on the seasonal pattern of microbial community successions, metabolic characteristics of the functional microorganism in Daqu, formation of flavors compound and also environment influence factor. It sums up all the present main strategies and studies on regulating season fluctuation in daqu. Nowadays, as for why microorganisms would have seasonal variation we don’t know about it much and the omics technology is mainly correlated with no way of validating causation, more exact controlling technologies or standardized procedures in industry production. In addition we can do Multi omics integration analysis, target regulation of certain community, smart improvement process in the future So as to reduce the difference in quality among different batches, stabilize seasonally fluctuating daqu in daqu production and provide theoretical basis for standardized and intelligent production of baijiu.

## Data Availability

No new data were created or analyzed in this study.

## References

[1] Tan Y., Zhong H., Zhao D., Du H., Xu Y. (2019). Succession Rate of Microbial Community Causes Flavor Difference in Strong-Aroma Baijiu Making Process. Int. J. Food Microbiol..

[2] Wei Y., Zou W., Shen C., Yang J. (2020). Basic Flavor Types and Component Characteristics of Chinese Traditional Liquors: A Review. J. Food Sci..

[3] Fu G., Cai W., Dong B., Wan Y., Pan F., Zheng F., Chen Y., Deng M., Huang B. (2022). Effects of Bio-Augmented Daqu on Microbial Community, Aroma Compounds and Physicochemical Parameters of Fermented Grains during the Brewing of Chinese Special-Flavor Baijiu. J. Sci. Food Agric..

[4] Xia Y., Zhu M., Du Y., Wu Z., Gomi K., Zhang W. (2022). Metaproteomics Reveals Protein Composition of Multiple Saccharifying Enzymes in Nongxiangxing Daqu and Jiangxiangxing Daqu under Different Thermophilic Temperatures. Int. J. Food Sci. Technol..

[5] Gong L., Qin S., Zheng X., Zhao J., Liu M., Zhao M. (2025). Traceability between Microbial Community and Environmental Microbial Community in Maotai-Flavor Daqu. Food Chem. X.

[6] Wang Y., Gai J., Hou Q., Zhao H., Shan C., Guo Z. (2023). Ultra-High-Depth Macrogenomic Sequencing Revealed Differences in Microbial Composition and Function between High Temperature and Medium–High Temperature Daqu. World J. Microbiol. Biotechnol..

[7] Wang L., Shen Y., Chen B., Nie H., Gan L., Luo A., He Q., Zhong K., Gao H. (2025). Spatial Environments of Stack and Layer Shape the Quality of Chinese Sauce Aroma Daqu: Characteristics, Functions, and Relationships. Int. J. Food Microbiol..

[8] Zhu M., Zheng J., Xie J., Zhao D., Qiao Z.-W., Huang D., Luo H.-B. (2022). Effects of Environmental Factors on the Microbial Community Changes during Medium-High Temperature Daqu Manufacturing. Food Res. Int..

[9] Zhu Q., Chen L., Peng Z., Zhang Q., Huang W., Yang F., Du G., Zhang J., Wang L. (2022). Analysis of Environmental Driving Factors on Core Functional Community during Daqu Fermentation. Food Res. Int..

[10] Wang L., Cheng Y., Hu X., Huang Y. (2022). Analysis of Bacterial Diversity and Functional Differences of Jiang-Flavored Daqu Produced in Different Seasons. Front. Nutr..

[11] Yang L., Fan W., Xu Y. (2024). Effects of Storage Period and Season on the Microecological Characteristics of Jiangxiangxing High-Temperature Daqu. Food Res. Int..

[12] Fang C., Shi G., He Q., Xing S., Chen P., Shi F., Liu Z., Tang P., Lin L., Zhang C. (2025). Microbiomics and Machine Learning-Assisted Approaches Reveal Amino Acid Patterns in High-Temperature Daqu. Food Chem..

[13] Wen Z., Wei Y.-H., Han D.-Y., Song L., Zhu H.-Y., Guo L.-C., Chen S.-X., Lin B., He C.-J., Guo Z.-X. (2025). Deciphering the Role of Traditional Flipping Crafts in Medium-Temperature Daqu Fermentation: Microbial Succession and Metabolic Phenotypes. Curr. Res. Food Sci..

[14] Huang P., Ma S., Yi X., Zhou R., Wu C. (2025). Seasonal Changes Driving Shifts in Core Microbes of Nongxiangxing Daqu: A Integrated Multi-Omics Analysis and Deep Learning. Food Res. Int..

[15] Liu Y., Li H., Liu W., Ren K., Li X., Zhang Z., Huang R., Han S., Hou J., Pan C. (2024). Bioturbation Analysis of Microbial Communities and Flavor Metabolism in a High-Yielding Cellulase *Bacillus subtilis* Biofortified Daqu. Food Chem. X.

[16] Liang F., Zhong Z., Ma M., Hao J., Ma P., Wu J., Huang H., Che F., Wu Q., Xu Y. (2024). Effect of Storage on Microbioa and Enzyme Proteomic Profile of Low-Temperature Daqu. Food Biosci..

[17] Zheng Y., Liang F., Wu Y., Ban S., Huang H., Xu Y., Wang X., Wu Q. (2023). Unraveling Multifunction of Low-Temperature Daqu in Simultaneous Saccharification and Fermentation of Chinese Light Aroma Type Liquor. Int. J. Food Microbiol..

[18] Viktor M.J., Rose S.H., van Zyl W.H., Viljoen-Bloom M. (2013). Raw Starch Conversion by *Saccharomyces cerevisiae* Expressing *Aspergillus tubingensis* Amylases. Biotechnol. Biofuels.

[19] Cai W., Wang Y., Ni H., Liu Z., Liu J., Zhong J., Hou Q., Shan C., Yang X., Guo Z. (2021). Diversity of Microbiota, Microbial Functions, and Flavor in Different Types of Low-Temperature Daqu. Food Res. Int..

[20] Fan G., Sun B., Fu Z., Xia Y., Huang M., Xu C., Li X. (2018). Analysis of Physicochemical Indices, Volatile Flavor Components, and Microbial Community of a Light-Flavor Daqu. J. Am. Soc. Brew. Chem..

[21] Zhang Z., Ran X., Guo Z., Hou Q., Qu D., Wang C., Xu Y., Wang Y. (2025). Microbial Diversity, Functional Properties, and Flavor Characteristics of High-Temperature Daqu with Different Colors. Food Res. Int..

[22] Chen Y., Zou L., Wang L., Dong W., Feng Y., Yu X., Liu J., Zhang Y., Hu Y., Chen S. (2025). Microbial, Physicochemical, and Flavor Interactions in High-Temperature Sauce-Flavor Daqu. Biology.

[23] Deng L., Mao X., Liu D., Ning X.-Q., Shen Y., Chen B., Nie H.-F., Huang D., Luo H.-B. (2020). Comparative Analysis of Physicochemical Properties and Microbial Composition in High-Temperature Daqu with Different Colors. Front. Microbiol..

[24] Zhang Y., Xu J., Jiang Y., Niu J., Chen X., Han B.-Z. (2022). Microbial Characteristics and Metabolite Profiles of High-Temperature Daqu in Different Maturation Stages. World J. Microbiol. Biotechnol..

[25] Cao L., Sun H., Wang Y., Wei Z., Zhang J., Wang Y., Yan J., Zhu Y., Cheng N., He S. (2025). Comparative Analysis of Metagenomics between High- and Medium-Temperature Daqu, and Microbial Succession in Jiang-Nong Jianxiang Baijiu Fermentation. BMC Genom..

[26] Nie X., Jia X., Zhu K., Ling Z., Chen H., Xie J., Ao Z., Song C., Shen C., Zhu C. (2024). Dynamic Changes and Potential Correlations between Microbial Diversity and Volatile Flavor Compounds in Chinese Medium-Temperature Daqu during Manufacturing. Molecules.

[27] Pan Q., Huang J., Zhang S., Qin H., Dong Y., Wang X., Mu Y., Tang H., Zhou R. (2023). Synergistic Effect of Biotic and Abiotic Factors Drives Microbiota Succession and Assembly in Medium-Temperature Daqu. J. Sci. Food Agric..

[28] Wen Z., Han P.-J., Han D.-Y., Song L., Wei Y.-H., Zhu H.-Y., Chen J., Guo Z.-X., Bai F.-Y. (2024). Microbial Community Assembly Patterns at the Species Level in Different Parts of the Medium Temperature Daqu during Fermentation. Curr. Res. Food Sci..

[29] Chen L., Peng Q., Chen Y., Che F., Chen Z., Feng S. (2025). Analysis of Dominant Microorganisms and Core Enzymes in Qingke Baijiu Daqu by High-Throughput Sequencing and Proteomics. Food Res. Int..

[30] Que P., Yao S., Hu J., Hu F., Wang D., Huang W. (2025). Associations of Microbial Communities with Metabolites and Enzyme Activities in Different Colors of High-Temperature Daqu. World J. Microbiol. Biotechnol..

[31] Xing S., Shi G., Lu J., Fang C., Li C., Yuan S., Shi F., Lin L., Zhang C. (2025). The Discrepancy in Amino Acids within High-Temperature Daqu: A Novel Metabolic Marker for the Quality Evaluation of Daqu. Food Chem..

[32] Yan C., Huang Z., Tu R., Zhang L., Wu C., Wang S., Huang P., Zeng Y., Shi B. (2025). Revealing the Differences in Microbial Community and Quality of High-Temperature Daqu in the Southern Sichuan-Northern Guizhou Region. Foods.

[33] Zhen P., Cao M., Jiang J., Yue K. (2025). Unveiling Microgeographical and Seasonal Variation in the Microbiome and Proteome of Fen-Flavored Daqu. Food Biosci..

[34] He M., Jin Y., Liu M., Yang G., Zhou R., Zhao J., Wu C. (2023). Metaproteomic Investigation of Enzyme Profile in Daqu Used for the Production of Nongxiangxing Baijiu. Int. J. Food Microbiol..

[35] Zhang Z., Fan H., Yu Z., Luo X., Zhao J., Wang N., Li Z. (2024). Metagenomics-Based Gene Exploration and Biochemical Characterization of Novel Glucoamylases and α-Amylases in Daqu and Pu-Erh Tea Microorganisms. Int. J. Biol. Macromol..

[36] Zhao Y., Han Y., Cao R., Xu Y., Mu X. (2025). Multiomics Insights into Microbiome Succession throughout Medium-Temperature Daqu Fermentation. ACS Food Sci. Technol..

[37] Liu P., Zhang L., Du X., Zhao J., Gao G., Zhang X. (2019). Dynamic Analysis of Physicochemical and Biochemical Indices and Microbial Communities of Light-Flavor Daqu during Storage. J. Am. Soc. Brew. Chem..

[38] Niu J., Li W., Du B., Wu Y., Lang Y., Sun B., Sun W., Li X. (2024). Temporal Heterogeneity of Microbial Communities and Flavor Metabolism during Storage of High-Temperature Daqu. Food Chem..

[39] Shi W., Chai L.-J., Zhao H., Song Y.-N., Mei J.-L., He Y.-X., Lu Z.-M., Zhang X.-J., Yang B., Wang S.-T. (2024). Deciphering the Effects of Different Types of High-Temperature Daqu on the Fermentation Process and Flavor Profiles of Sauce-Flavor Baijiu. Food Biosci..

[40] Ye H., Wang J., Shi J., Du J., Zhou Y., Huang M., Sun B. (2021). Automatic and Intelligent Technologies of Solid-State Fermentation Process of Baijiu Production: Applications, Challenges, and Prospects. Foods.

[41] Cao R., Zhou Q., Ma Y., Yan X., Li A., Du H., Xu Y. (2025). Multimodal Integration: Mechanisms of Temperature Dynamics and Quality Formation Critical Period in Daqu. Food Res. Int..

[42] Huang Y., Li D., Mu Y., Zhu Z., Wu Y., Qi Q., Mu Y., Su W. (2023). Exploring the Heterogeneity of Community and Function and Correspondence of “Species-Enzymes” among Three Types of Daqu with Different Fermentation Peak-Temperature via High-Throughput Sequencing and Metagenomics. Food Res. Int..

[43] Zhang P., Liu Y., Li H., Wang S., Li X., Xu L., Zhang Z., Huang R., Han S., Pan C. (2024). Bioturbation Effect of High—Yield Pyrazine Strain on the Microbial Community and Flavour Metabolites of Fortified Daqu. LWT Food Sci. Technol..

[44] Zhao M., Han C., Xue T., Ren C., Nie X., Jing X., Hao H., Liu Q., Jia L. (2025). Establishment of a Daqu Grade Classification Model Based on Computer Vision and Machine Learning. Foods.

[45] Zhu Y., Chen H., Zeng X., Jiang X., Su Y., Cang Y., Long W., Lan W., Fu H., She Y. (2025). Rapid Visual Authentication of High-Temperature Daqu Baijiu Using Porphyrin Signal Amplification and Smartphone-Based Cloud Machine Learning. Spectrochim. Acta A Mol. Biomol. Spectrosc..

[46] Izydorczyk G., Skrzypczak D., Mironiuk M., Mikula K., Samoraj M., Gil F., Taf R., Moustakas K., Chojnacka K. (2024). Lignocellulosic Biomass Fertilizers: Production, Characterization, and Agri-Applications. Sci. Total Environ..

[47] Jiang X., Peng Z., Liu H., Zhang L., Zhang J. (2025). Assembly of a Lignocellulose-Degrading Synthetic Community from the Strong-Flavor Daqu by a Stepwise Method. Food Res. Int..

[48] Luo H., Akkermans S., Van Impe J.F.M. (2025). Demystifying Food Flavor: Flavor Data Interpretation through Machine Learning. Food Chem..

[49] Ma S., Luo H., Zhao D., Qiao Z., Zheng J., An M., Huang D. (2021). Environmental Factors and Interactions among Microorganisms Drive Microbial Community Succession during Fermentation of Nongxiangxing Daqu. Bioresour. Technol..

[50] Mu Y., Huang J., Zhou R., Zhang S., Qin H., Tang H., Pan Q., Tang H. (2023). Bioaugmented Daqu-Induced Variation in Community Succession Rate Strengthens the Interaction and Metabolic Function of Microbiota during Strong-Flavor Baijiu Fermentation. LWT Food Sci. Technol..

[51] Xu T., Zhu A., Zhong S., Wang Y., Wang C., Zhang J., Chen Y., Wang R., Zhang C. (2025). Physicochemical Properties and Bioturbation Analysis of High-Temperature Daqu by Functional Microflora. Front. Microbiol..

[52] Yao Y., Luo Y., Wu M., Gao L., Zhang J., Jiang Z., Jiang X., Xia X. (2025). Contribution of Moisture to Functional Microbial Succession and Functional Expression in Medium-High Temperature Daqu. Food Biosci..

[53] Zhu Q., Chen L., Pu X., Du G., Yang F., Lu J., Peng Z., Zhang J., Tu H. (2023). The Differences in the Composition of Maillard Components between Three Kinds of Sauce-Flavor Daqu. Fermentation.

[54] Mu Y., Huang J., Zhou R., Zhang S., Qin H., Tang H., Pan Q., Tang H. (2023). Characterization of the Differences in Aroma-Active Compounds in Strong-Flavor Baijiu Induced by Bioaugmented Daqu Using Metabolomics and Sensomics Approaches. Food Chem..

[55] Qu D., Wang Y., Cao L., Hou Q., Liu Z., Zhong J., Guo Z. (2025). Low-Temperature Daqu Types Differentially Shape Microbial and Metabolic Profiles in Fermented Grains: Insights from Metagenomics and Flavor Omics. Food Biosci..

[56] Huang P., Jin Y., Liu M., Peng L., Yang G., Luo Z., Jiang D., Zhao J., Zhou R., Wu C. (2023). Exploring the Successions in Microbial Community and Flavor of Daqu during Fermentation Produced by Different Pressing Patterns. Foods.

[57] Liu W., Liu G., Zhang R., Zheng L., Lu Z., Zhang X., Wang S., Shen C., Shi J., Xu Z. (2024). Metagenomics Unveils the Differences in the Functions of Microbial Community of Medium-Temperature Daqu before and after Maturation. Chin. J. Biotechnol..

[58] Liu Y., Zhang P., Li H., Yang C., Li M., Huang R., Han S., Hou J., Pan C. (2025). Microbial Origin of Fermented Grains in Different Fermentation Stages of Taorong-Type Baijiu. Food Res. Int..

[59] Tong W., Wang S., Yang Y., Huang Z., Li Y., Huang D., Luo H., Zhao L. (2023). Insights into the Dynamic Succession of Microbial Community and Related Factors of Vanillin Content Change Based by High-Throughput Sequencing and Daqu Quality Drivers. Foods.

[60] Tanaka M., Gomi K. (2021). Induction and Repression of Hydrolase Genes in *Aspergillus oryzae*. Front. Microbiol..

[61] Tang Q., Chen X., Huang J., Zhang S., Qin H., Dong Y., Wang C., Wang X., Wu C., Jin Y. (2023). Mechanism of Enhancing Pyrazines in Daqu via Inoculating *Bacillus licheniformis* with Strains Specificity. Foods.

[62] He G., Dong Y., Huang J., Wang X., Zhang S., Wu C., Jin Y., Zhou R. (2019). Alteration of Microbial Community for Improving Flavor Character of Daqu by Inoculation with *Bacillus velezensis* and *Bacillus subtilis*. LWT Food Sci. Technol..

[63] Li W., Fan G., Fu Z., Wang W., Xu Y., Teng C., Zhang C., Yang R., Sun B., Li X. (2019). Effects of Fortification of Daqu with Various Yeasts on Microbial Community Structure and Flavor Metabolism. Food Res. Int..

[64] Chen P., Zhang D., Mkunga J.J., Zhai W., Shan C., Yang X., Cai W. (2025). Exploration of Core Microorganisms and Synthetic Microbial Communities in Low-Temperature Daqu. Microorganisms.

[65] Kang J., Zheng X., Yang X., Li H., Cheng J., Fan L., Zhao H., Xue Y., Ding Z., Han B. (2022). Contrasting Summer versus Winter Dynamic Microbial Communities and Their Environmental Driving Factors in the Solid-State Saccharification Process of Fuyu-Flavor Baijiu. Food Res. Int..

[66] He Y., Qiao M., Zhang H., Xiao D., Guo X. (2025). Microbial Community, Metabolic, and Flavor Differences among High-Temperature Daqu with Varying Douchi Aroma Intensities: A Comprehensive Metagenomic and Metabolomic Analysis. Food Chem. X.

[67] Wang Y., Lei Y., Zhang J., Hou Q., Guo Z. (2025). Differential Characterization of Medium-Temperature Daqu from Different Geographical Regions: Bacterial Profile, Color, Taste, and Flavor. World J. Microbiol. Biotechnol..

[68] Zhu C., Cheng Y., Shi Q., Ge X., Yang Y., Huang Y. (2023). Metagenomic Analyses Reveal Microbial Communities and Functional Differences between Daqu from Seven Provinces. Food Res. Int..

[69] Dong W., Yu X., Wang L., Zou M., Ma J., Liu J., Feng Y., Zhao S., Yang Q., Hu Y. (2024). Unveiling the Microbiota of Sauce-Flavor Daqu and Its Relationships with Flavors and Color during Maturation. Front. Microbiol..

[70] Zhang Y., Kang J., Han B.-Z., Chen X. (2023). Wheat-Origin *Bacillus* Community Drives the Formation of Characteristic Metabolic Profile in High-Temperature Daqu. LWT Food Sci. Technol..

[71] Jiang X., Peng Z., Zhu Q., Zheng T., Liu X., Yang J., Zhang J., Li J. (2023). Exploration of Seasonal Fermentation Differences and the Possibility of Flavor Substances as Regulatory Factors in Daqu. Food Res. Int..

[72] Li B., Tang J., Zhu C., Gong J., Wu Q., Yang Y., Ge X., Wei J., Huang Y. (2026). Analyzing the Metabolic Mechanisms and Regulation of Flavor Compounds in Baijiu from a Microbial Sociology Perspective. Food Biosci..

[73] Ma S., Zhang Y., Zhou Y., Duan Z., Huang D., Huang P., Niu J., Cheng W., Wu C. (2026). Ecological Processes in Microbial Community Time Series during Sauce-Flavor Baijiu Fermentation. Food Res. Int..

[74] Pan F., Qiu S., Lv Y., Li D. (2023). Exploring the Controllability of the Baijiu Fermentation Process with Microbiota Orientation. Food Res. Int..

[75] Wang H., Chen Z., Qing H., Huang M., Xue G., Lu X., Che Y., Dong Y., Zhang S., Yu J. (2025). Seasonal Influence on the Microbial Diversity and Flavor Substances in the Strong Flavor Daqu Fermentation. Food Chem. Mol. Sci..

[76] Niu J., Yang S., Shen Y., Cheng W., Li H., Sun J., Huang M., Sun B. (2022). What Are the Main Factors That Affect the Flavor of Sauce-Aroma Baijiu. Foods.

[77] Yang S., Duan J., Lv S., Xu L., Li H. (2022). Revealing the Changes in Compounds When Producing Strong-Flavor Daqu by Statistical and Instrumental Analysis. Fermentation.

[78] Fu G., Deng M., Chen Y., Chen Y., Wu S., Lin P., Huang B., Liu C., Wan Y. (2021). Analysis of Microbial Community, Physiochemical Indices, and Volatile Compounds of Chinese Te-Flavor Baijiu Daqu Produced in Different Seasons. J. Sci. Food Agric..

[79] Tang Q., Zhang Y., Huang J., Zhou R. (2025). Directed Regulation of High-Temperature Daqu Microbiota and Metabolites Using Synthetic Communities. Food Microbiol..

[80] Yang J.-G., Dou X., Han P.-J., Bai F.-Y., Zhou J., Zhang S.-Y., Qin H., Ma Y.-Y. (2017). Microbial Diversity in Daqu During Production of Luzhou-Flavored Liquor. J. Am. Soc. Brew. Chem..

[81] Ban S., Shen Y., Cheng W., Chen B., Zhang Y., Nie H., Wang S., Xu Y., Wu Q. (2024). Community Dynamics and Assembly Is Driven by Environmental Microbiota Mediated by Spatiotemporal Distribution: The Case of Daqu Fermentation. Int. J. Food Microbiol..

[82] Zeng H., Jiang X., Wang Z., Zeng X., Xin B., Wang Y., Zhang X., Yang H., Qiao J., Dong R. (2021). Environmental and Physicochemical Characterization and Fungal Community of Two Batches of Chinese Luzhou-Flavored Daqu. J. Am. Soc. Brew. Chem..

[83] Wang Z., Wang S., Liao P., Chen L., Sun J., Sun B., Zhao D., Wang B., Li H. (2022). HS-SPME Combined with GC-MS/O to Analyze the Flavor of Strong Aroma Baijiu Daqu. Foods.

[84] Wang X.-D., Qiu S.-Y., Li P., Ban S.-D. (2019). Analysis of Microbial Community Structure in Traditional and Automated Moutai-Flavor Daqu. J. Am. Soc. Brew. Chem..

[85] Zhou Y., Liu N., Li Z., Peng K., Wang C., Xia Y., Huang D., Luo H. (2025). The Rate of Change in Temperature and Humidity during the Temperature Increase Period Determines the Microbial Community Structure and Function of Medium-High temperature Daqu. Food Biosci..

[86] Ge D., Cai W., Guo Z., Liu Z., Xu Y., Shan C. (2024). Comparative Analysis of Microbial Community Structure in the Peel and Core of Houhuo Low-Temperature Daqu. Food Biosci..

[87] Luo S., Zhang Q., Yang F., Lu J., Peng Z., Pu X., Zhang J., Wang L. (2022). Analysis of the Formation of Sauce-Flavored Daqu Using Non-Targeted Metabolomics. Front. Microbiol..

[88] Liu Z., Wang X., Qin H., Chen Y., Xia L., Wang X., Lai Y., Li G. (2025). Research Progress of the Maillard Reaction Process Monitoring. Chin. Chem. Lett..

[89] Zhu C., Cheng Y., Zuo Q., Huang Y., Wang L. (2022). Exploring the Impacts of Traditional Crafts on Microbial Community Succession in Jiang-Flavored Daqu. Food Res. Int..

[90] Li X., Liao B., Wang X., Dong W., Li J., Hu Y., Li Y., Peng N., Zhao S. (2025). Non-*Saccharomyces* Yeasts Enhance Yield and Flavor in Industrial Xiaoqu Light-Flavor Baijiu Production. Food Res. Int..

[91] Deng N., Du H., Xu Y. (2020). Cooperative Response of *Pichia kudriavzevii* and *Saccharomyces cerevisiae* to Lactic Acid Stress in Baijiu Fermentation. J. Agric. Food Chem..

[92] Li R.-R., Xu M., Zheng J., Liu Y.-J., Sun C.-H., Wang H., Guo X.-W., Xiao D.-G., Wu X.-L., Chen Y.-F. (2022). Application Potential of Baijiu Non-*Saccharomyces* Yeast in Winemaking Through Sequential Fermentation with *Saccharomyces cerevisiae*. Front. Microbiol..

[93] Wei J., Du H., Xu Y. (2024). Revealing the Key Microorganisms Producing Higher Alcohols and Their Assembly Processes during Jiang-Flavor Baijiu Fermentation. Food Biosci..

[94] Zhang G., Xiao P., Xu Y., Li H., Li H., Sun J., Sun B. (2023). Isolation and Characterization of Yeast with Benzenemethanethiol Synthesis Ability Isolated from Baijiu Daqu. Foods.

[95] Huang Q., Liu Y., He Z., Mao Y., Wu H., Tian L., Xiang S., Long L., Li Y., Guan T. (2024). Environmental Temperature Variations Drive the Changes of Microbial Communities to Affect Baijiu Flavor Quality: Case Study of Qingxiangxing Baijiu. Food Biosci..

[96] Chai L.-J., Zhang J.-Y., Gao T., Zhang L.-Y., Zhang X.-J., Lu Z.-M., Shi J.-S., Chen X., Xu Z.-H. (2024). Seasonality Shapes the Microbiota and Metabolome of Strong-Flavor Baijiu during Fermentation and Affects Its Flavor Characteristics. Food Biosci..

[97] Li C., Yang F., Han Y., Yang C., Qin X., Zheng H., Chen L., Lu J., Zhang C., Lu F. (2025). Aldehyde Metabolism in Maotai-Flavor Baijiu: Insights from Integrated Metagenomic and Metaproteomic Analyses. Food Res. Int..

[98] Shi W., Chai L.-J., Fang G.-Y., Mei J.-L., Lu Z.-M., Zhang X.-J., Xiao C., Wang S.-T., Shen C.-H., Shi J.-S. (2022). Spatial Heterogeneity of the Microbiome and Metabolome Profiles of High-Temperature Daqu in the Same Workshop. Food Res. Int..

[99] Wang Y., Hou Q., Ni H., Tian L., Liu J., Zhou J., Guo Z. (2024). Multi-Method Joint Analysis Reveals Differences in the Quality and Microbial Composition of High-Temperature and Medium–High-Temperature Daqu. LWT Food Sci. Technol..

[100] Xu Y., Sun B., Fan G., Teng C., Xiong K., Zhu Y., Li J., Li X. (2017). The Brewing Process and Microbial Diversity of Strong Flavour Chinese Spirits: A Review. J. Inst. Brew..

[101] Zhao J., Yang Y., Chen L., Zheng J., Lv X., Li D., Fang Z., Shen C., Mallawaarachchi V., Lin Y. (2023). Quantitative Metaproteomics Reveals Composition and Metabolism Characteristics of Microbial Communities in Chinese Liquor Fermentation Starters. Front. Microbiol..

[102] Pang Z., Li W., Hao J., Xu Y., Du B., Zhang C., Wang K., Zhu H., Wang H., Li X. (2023). Correlational Analysis of the Physicochemical Indexes, Volatile Flavor Components, and Microbial Communities of High-Temperature Daqu in the Northern Region of China. Foods.

[103] Ren T., Su W., Mu Y., Qi Q., Zhang D. (2023). Study on the Correlation between Microbial Communities with Physicochemical Properties and Flavor Substances in the Xiasha Round of Cave-Brewed Sauce-Flavor Baijiu. Front. Microbiol..

[104] Xu Y., Qiao X., He L., Wan W., Xu Z., Shu X., Yang C., Tang Y. (2024). Airborne Microbes in Five Important Regions of Chinese Traditional Distilled Liquor (Baijiu) Brewing: Regional and Seasonal Variations. Front. Microbiol..

[105] Li Y., Qiao H., Zhang R., Zhang W., Wen P. (2023). Microbial Diversity and Volatile Flavor Compounds in Tibetan Flavor Daqu. Foods.

[106] Cryan J.F., O’Riordan K.J., Cowan C.S.M., Sandhu K.V., Bastiaanssen T.F.S., Boehme M., Codagnone M.G., Cussotto S., Fulling C., Golubeva A.V. (2019). The Microbiota-Gut-Brain Axis. Physiol. Rev..

[107] Zheng T., Zhang Q., Wu Q., Li D., Wu X., Li P., Zhou Q., Cai W., Zhang J., Du G. (2022). Effects of Inoculation with *Acinetobacter* on Fermentation of Cigar Tobacco Leaves. Front. Microbiol..

[108] Xiong X., Hu Y., Yan N., Huang Y., Peng N., Liang Y., Zhao S. (2014). PCR-DGGE Analysis of the Microbial Communities in Three Different Chinese “Baiyunbian” Liquor Fermentation Starters. J. Microbiol. Biotechnol..

[109] Fusco V., Chieffi D., Fanelli F., Montemurro M., Rizzello C.G., Franz C.M.A.P. (2023). The *Weissella* and *Periweissella* Genera: Up-to-Date Taxonomy, Ecology, Safety, Biotechnological, and Probiotic Potential. Front. Microbiol..

